# Alkaline Membranes toward Electrochemical Energy Devices: Recent Development and Future Perspectives

**DOI:** 10.1021/acscentsci.3c00597

**Published:** 2023-07-14

**Authors:** Wanjie Song, Xin Zhang, Cui Yang, Zhengjin Yang, Liang Wu, Xiaolin Ge, Tongwen Xu

**Affiliations:** † Key Laboratory of Precision and Intelligent Chemistry, Collaborative Innovation Centre of Chemistry for Energy Materials, School of Chemistry and Material Science, University of Science and Technology of China, Hefei 230026, P.R. China

## Abstract

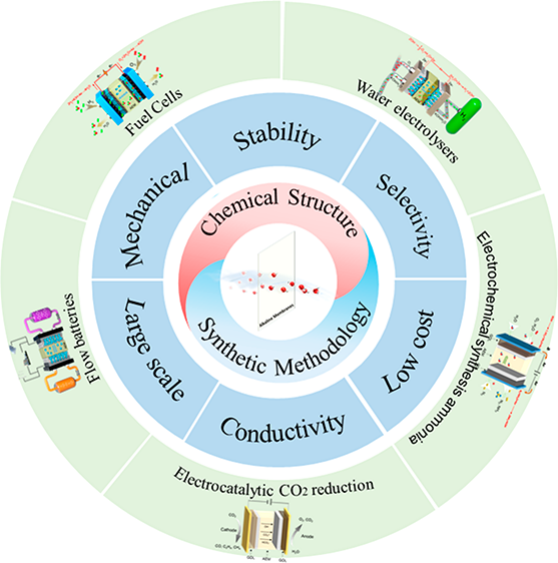

Anion-exchange membranes (AEMs) that can selectively transport OH^–^, namely, alkaline membranes, are becoming increasingly crucial in a variety of electrochemical energy devices. Understanding the membrane design approaches can help to break through the constraints of undesired performance and lab-scale production. In this Outlook, the research progress of alkaline membranes in terms of backbone structures, synthesis methods, and related applications is organized and discussed. The evaluation of synthesis methods and description of membrane stability enhancement strategies provide valuable insights for structural design. Finally, to accelerate the deployment of relevant technologies in alkaline media, the future priority of alkaline membranes that needs to be addressed is presented from the perspective of science and engineering.

## Abstract 2

This Outlook presents the advances and challenges for alkaline membranes toward electrochemical energy devices.

## Introduction

Anion-exchange membranes (AEMs) are an essential class of membrane-like functional polymer materials, consisting of a polymer matrix, positively charged groups, and mobile counteranions. To tackle complex environmental problems and energy crises, it is widely used in separation and purification devices as well as electrochemical components,^1,2^ wherein alkaline membranes are a class of AEMs used in alkaline media energy technologies for conducting OH^–^ anions. The alkaline membrane-based energy technologies, such as fuel cells,^3^ water electrolyzers,^4^ flow batteries,^5^ CO_2_^6^ and N_2_^7^ electroreduction, and other electrochemical technologies, have attracted extensive attention. Generally, the proton-exchange membrane (PEM) technologies feature higher power density, faster H_2_ production rate, and improved cell efficiency. However, it has to be noted that the components, including platinum group metal catalysts (determined by the acidic working conditions), membranes, and bipolar plates, have relatively high costs. In sharp contrast, the superior conversion reactions at the electrodes in alkaline media promote the use of non-noble metal catalysts. Furthermore, the less corrosive environment reduces the cell component’s cost, increases the catalyst durability, and prolongs the lifespan of cell devices. These advantages emphasized the importance of AEM technologies.

Acting as charge carrier conductors and electrode separators, alkaline membranes are critical components in electrochemical processes. Besides, they are highly correlated with cell performance and durability. In view of energy technology developments, alkaline membranes have triggered increasing attention in academic as well as industrial fields. Thousands of publications have focused on alkaline membranes, mainly alkaline membrane-based fuel cell technology. Proverbially, two key elements (conductivity and stability) restrict the development and application of alkaline membranes. With regard to improving conductivity, the construction of microphase separation^8^ to facilitate ion conduction rates and regulation of micropores^9^ to reduce ion conduction hindrance are two popular approaches. With these effective strategies in mind, significant progress has been made. So far, the conductivity has gone from less than 10 mS cm^–1^ to exceeding 100 mS cm^–1^. Furthermore, the development of aryl ether-free backbone and stable N-heterocyclic ammonium groups raised the membranes’ alkaline stability to a new level.^10^ Currently, the membranes’ ex-alkaline stability exceeds 2000 h. Unfortunately, a limited number of commercially available alkaline membranes exist, and even fewer can be deployed in energy technologies. This is mainly due to the unsustainable robust membrane stability and unbefitting scale production methods.

In this Outlook, since multiple membrane structures have been developed, we systematically collate different types of polymeric backbones with respect to their structures and preparation, summarize the strategies for improving alkaline stability, and highlight achievements that bring significant effectiveness to corresponding electrochemical energy technology. Furthermore, we focus on the relationship between membrane stability and cell durability and point out future research directions for molecular structure design approaches.

## 2 Types of Alkaline Membranes

### 2.1 Poly(arylene ether)-Based Alkaline Membranes

In the early period of alkaline membrane research, low-cost and easily available engineering plastics such as poly(arylene ether sulfone) (PAES), poly(arylene ether ketone (PAEK), partially fluorinated poly(arylene ether) (FPAE), and poly(2,6-dimethyl-1,4-phenylene oxide) (PPO) are mainly used as backbones. These backbones are featured with aryl-ether bonds and the possible existence of some electron-withdrawing groups (e.g., sulfone, ketone, or perfluoro groups). The following summarizes the general functional modification methods and performance improvement strategies of these poly(aryl ether) type alkaline membranes.

**Figure 1:**
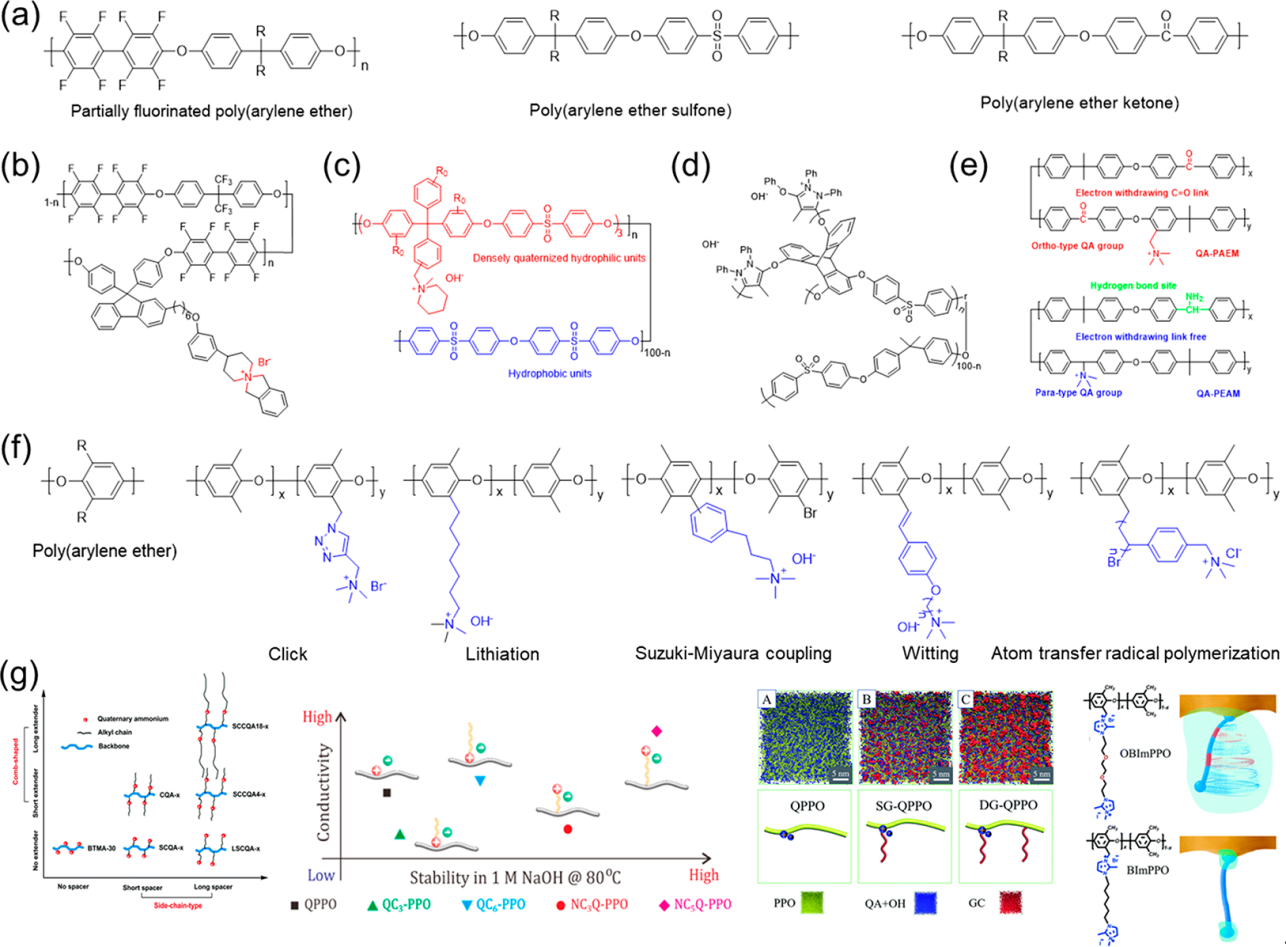
(a) Representative chemical structures of FPAE, PAES, PAEK. (b–e) Representative strategies for membrane stability enhancement. (f) Representative chemical structures of PPO and benzyl-free PPO-based alkaline membranes.^22−26^ (g) Effects of different side chain types on membrane stability.^27−30^ Reproduced with permission from refs (27−30). Copyright 2018 Royal Society of Chemistry, Copyright 2017 American Chemical Society, and Copyright 2018 Royal Society of Chemistry.

The typical chemical structures of poly(arylene ether)-based membrane backbones are plotted in Figure 1a. For preparing the cation-functionalized poly(arylene ether) membranes, the chloromethylation/bromination-quaternization route is a conventional functional modification, and the bromination–quaternary amination route is a viable alternative scheme. Other preparation methods are nucleophilic substitution reactions between activated fluorine and amine or dihalide and bisphenol monomers.

Notably, these backbones are remarkably alkaline stable in highly alkaline environments, but due to electrostatic interactions, the attached ionic groups neighboring the aryl-ether bonds attract more OH^–^ to close approach, which will attack and break the C–O bond. Ramani et al.^11^ used a 2D NMR technique to study the chemical degradation pathway of the quaternary amine (QA) functionalized PAES that was exposed to alkaline media. They demonstrated that the polymer backbone was degraded via quaternary carbon and ether hydrolysis under the trigger of QA. Further, Ein-Eli et al.^12^ investigated that the electron-withdrawing sulfone group accelerated backbone degradation by reducing the activation energy barrier of C–O bond cleavage. Similarly, the PAEK-based backbone would also have electron-withdrawing properties. Severe molecular weight degradation was observed by immersing the membrane in 1 M NaOH solution at 60 or 80 °C for 48 h.^13^

To deal with this instability, several possible strategies have been proposed. (1) Separating the QA group from the backbones with the use of pendant groups may alleviate the attack of OH^–^ to some extent.^14,15^ Zhang et al.^16^ grafted flexible side chains through the Williamson reaction and adopted more chemically stable N-spirocyclic QA groups to prepare the alkaline membrane. The PAENQA-1.0 showed a 6% conductivity degradation after 480 h in 1 M KOH at 80 °C (Figure 1b). Hence, keeping hydrophilic ionic groups as far away as possible from electron-withdrawing groups in the backbone should be borne in mind for structural design. (2) Reducing the number of electron-withdrawing groups from the membrane’s backbone is available. Introducing copolymerized block components, such as the most exciting example of a block copolymer, seems promising (Figure 1c). Compared with the random copolymer, block copolymer in the polymer backbone is a successful approach to construct well-defined hydrophilic–hydrophobic phase-separated morphology because it maximizes the use of water molecules without sacrificing physicochemical properties.^17^ Research shows that a microblock membrane has advantages over random membrane both in anion conductivity and in alkali stability.^18^ Bae et al.^19^ found that the hydrophobic block affected anion conductivity, while the hydrophilic block mainly affected alkali stability. Considering that both the hydrophobic (rigidity and hydrophobicity) and hydrophilic (flexibility and hydrophilicity) block components affect the membrane properties, a cautious and delicate structural design is required to produce well-defined phase separations. (3) Proverbially, cross-linking is an efficacious method to fabricate more stable membranes, especially chemical and ionic cross-linking. But the dense cross-linked network may hinder OH^–^ transport to some extent. To simultaneously reduce conduction barriers and increase durability, Swager et al.^20^ proposed an ionic cross-linking strategy, as shown in Figure 1d. They reported a series of pyrazolium cross-linked poly(triptycene ether sulfone) membranes. The cross-linked membranes constructed ionic highways along charge-delocalized pyrazolium cations and homoconjugated triptycene. An optimized PX75-T50 showed improved conductivity of 111.6 mS cm^–1^ at 80 °C. It also displayed an enhanced conductivity retention capacity (relatively 24% conductivity decrease after 720 h in 1 M KOH at 80 °C) due to the resonance stabilization. Generally, these strategies have improved conductivity and stability to some extent, but the detriment of electron-withdrawing is not solved fundamentally. He et al.^21^ alleviated the critical trigger for aryl ether cleavage by converting the C=O link (electron-withdrawing) on the PAEK backbone into C–NH_2_ (electron-donating) (Figure 1e). The resulting membrane demonstrated excellent stability without backbone degradation under harsh conditions (4 M KOH, 80 °C, 400 h). Although some progress has been made, the unsatisfied membrane performance, including lower OH^–^ conductivity (<100 mS cm^–1^ at 80 °C) and poor alkaline stability (conductivity retention <90% after 1000 h in 1 M KOH, 80 °C), limits its practical application.

The use of PPO backbones avoids the adverse effects of electron-withdrawing groups (Figure 1f). To further optimize the membrane stability, attention was focused on benzyl elimination and side-chain engineering.

The presence of a positively charged nitrogen cation at the activated benzylic position accelerates the nucleophilic attack of OH^–^. To avoid cation-induced degradation, some synthesis attempts including click reaction,^22^ benzylic lithiation,^23^ Suzuki–Miyaura coupling reaction,^24^ Witting reactions,^25^ and atom transfer radical polymerization^26^ have been exploited. Moreover, through the systematic study of side chain engineering (Figure 1g), it is found that the length, location, and stiffness of the side chains (in the middle of the ionic group, at the end of the ionic group, away from the ionic group) affect membrane stability by influencing the steric hindrance of functional groups and membrane hydration. Simultaneously, the density and distribution of ionic moieties along the side chains also affect membrane conductivity and stability. These practiced strategies improve the membrane stability to a certain extent and can provide guidance for the following alkaline membrane structure design.

In sum, the synthesis and modification of engineering plastics were the leading research directions of alkaline membranes over the past few years. Also, some new structures have been developed. But almost all aromatic backbone polymers consist of benzylic hydrogens or aryl ether bonds that were used as the basis for alkaline membranes. Although various strategies based on steric hindrance and electronic effects have been developed for preparing alkali stable membranes, the degradation risk of the aryl-ether-bond backbone cannot be fundamentally avoided, which inevitably leads to mechanical property loss and molecular weight decrease. Most poly(arylene ether)-based alkaline membranes still exhibit inferior performance in terms of essential indexes, including anion conductivity and alkali stability. Yet there are few poly(arylene ether)-based alkaline membranes with reported conductivity exceeding 100 mS cm^–1^ and alkali stability for longer than 1000 h, which limit its wide application in electrochemical devices. The low conductivity brings significant charge transfer resistance, resulting in poor application performance. Additionally, the membrane pinhole development or fracture propagation caused by membrane degradation is a fatal injury in the application of energy devices. Therefore, molecular engineering design to eliminate aryl ether bonds is urgent. Polymers free of aryl ether bonds have attracted more interest and have become research hotspots.

### 2.2 Polyrotaxane-Based and Polymers with Intrinsic Microporosity-Based Alkaline Membranes

**Figure 2:**
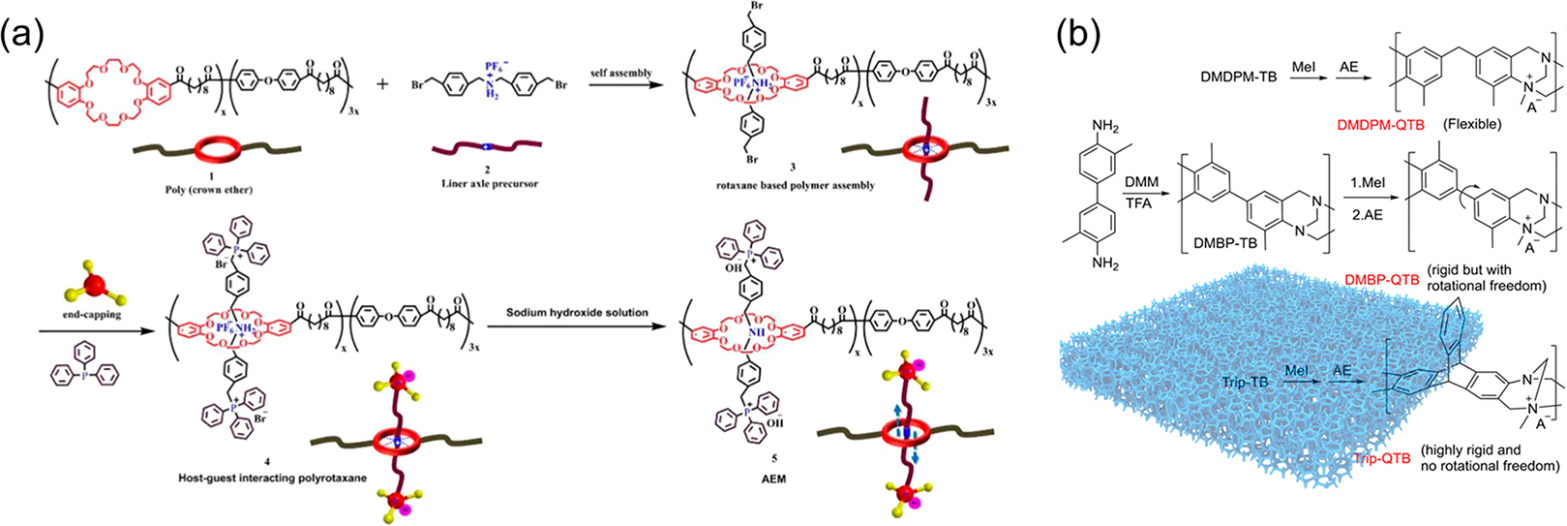
Synthetic route for (a) polyrotaxane^31^ and (b) polymers with intrinsic microporosity-based alkaline membranes.^9^ Reproduced with permission from refs (31) and (9). Copyright 2021 American Association for the Advancement of Science and Copyright 2020 John Wiley and Sons.

Despite promising achievements of microphase-separated membranes in promoting fast anion conducting, alkaline membranes still face lower conductivity due to their irregularly regulated morphology at the nanoscale and the finite wiggle space of covalently connected charged groups. For this purpose, our group pioneered the concept of molecular machines in membrane design.^31−33^ As presented in Figure 2a, the polyrotaxane membranes were developed by threading ionic linear guest into poly(crown ether) hosts through host–guest molecular interaction. As expected, the conductivity was remarkably enhanced (189 mS cm^–1^ at 90 °C and 0.68 mmol g^–1^). It is attributed to the increased solvation-shell fluctuations in inactive hydrated OH^–^ complexes around the function groups under the trigger of thermal and pH. Although the polyrotaxane-based membrane has made a significant breakthrough in conductivity, membrane stability remains a problem due to the existence of aryl ether bond.

In addition to low anion conductivity, detrimental dimensional stability is a non-negligible issue of alkaline membranes. The high swelling ratio (>20%) of current membranes encourages us to explore novel topologies of polymer materials. Research shows that microporous polymers may be a promising class of materials as the abundant free-volume holes within the membrane can provide water storage space without swelling. Our group proposed the concept of using confined subnanometer channels to prepare intrinsic microporosity membranes^9^ (Figure 2b). Fast ion transport is achieved by enhancing charge interactions in the confined subnanometer channels. The resulting membrane displayed a lower SR of 5% at high conductivity (164.4 mS cm^–1^, 80 °C). The implementation of this concept gets rid of the ill-defined and prone swell of ion channels. The functionalities of size sieving and high permeability endow the membrane with high ion conductivity and high selectivity, which make it show incredible charm in aqueous organic redox flow batteries. These ingenious design strategies have opened new doors for conductivity improvement. However, the application of this membrane type in alkaline media such as fuel cells and water electrolyzers needs to be further improved due to the unstable group, bridged bond, and poor gas barrier properties. Hence, efforts need to be devoted to membrane stability and suitable application scenarios.

### 2.3 Nitrogen-Conjugated Alkaline Membranes

Nitrogen-conjugated cationic polymers are attractive structures used for alkaline membranes due to their susceptibility to modification and π-conjugated delocalization. The conjugated structure can decrease the nucleophilic attack probability and improve the alkali resistance by reducing the positive charge density of cations and making the electron cloud delocalize.

**Figure 3:**
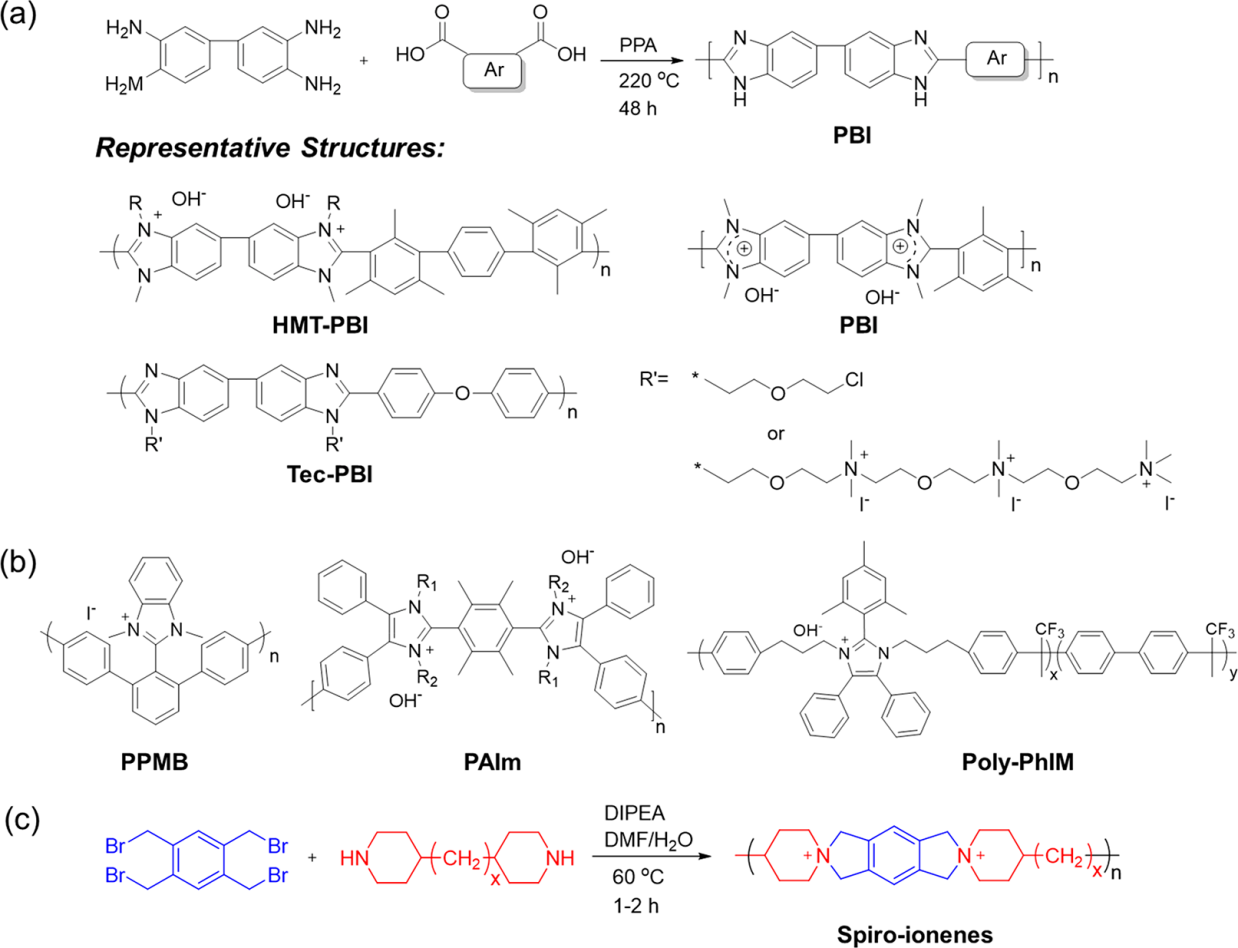
(a) Dehydration cyclization polycondensation to synthesize PBI and representative chemical structure of PBI-based alkaline membranes.^34,35,38^ (b) Representative chemical structure of poly(imidazole)-based alkaline membranes.^36,43,44^ (c) Cyclo-polycondensation to prepare spiro-ionene.^45^

Poly(benzimidazolium) (PBI) is a class of materials with benzimidazole rings in the backbone, usually prepared by polycondensation and cyclization of the corresponding diamines and carboxylic acids (Figure 3a). Ring-opening degradation is one of the major degradation pathways of methylated PBI, which is caused by OH^–^ attack on the C2-position.^34^ Strategies, such as increasing the electron density at the C2-position, extending the distance between the benzimidazole repeating units, and introducing steric hindrance around C2-position by way of proximal methyl groups, are adopted to reduce the OH^–^ attack probability.^35−37^ For instance, the HMT-PMBI membrane showed an improved membrane stability (6% chemical degradation after 168 h in 2 M NaOH, 80 °C).^35^ More importantly, it has become a commercial product named Aemion. The Tec-PBI-50 reported by He et al.^38^ (131.8 mS cm^–1^ at 80 °C and 78% conductivity retention for 672 h in 2 M KOH, 60 °C) demonstrated an excellent PPD of 1.16 W cm^–2^ in H_2_/O_2_ fuel cells. Additionally, acid-doped PBI membranes have aroused widespread research interest in high-temperature proton exchange membrane fuel cells and redox flow batteries.^39,40^ Excitingly, the KOH doped PBI membranes, called ion-solvating membranes, exhibited an unexpected effect in alkaline water electrolyzers.^41,42^ Recent research indicated that the KOH-doped PBI with the support of PTFE displayed a promising performance (1.8 A cm^–2^ at 1.8 V) and durability (operated for 1000 h).^41^ But all these research results indicated that there was no great progress in alkaline stability of cation-functionalized PBI membranes. The newly devised route is displayed in Figure 3b, such as Yamamoto coupling and Friedel–Crafts polycondensations, which significantly promoted the alkaline stability of poly(imidazole)-based alkaline membranes.^36,43,44^ The fabricated membranes exhibited admirable alkali stability in a harsher environment (10 M KOH, 80 °C). Additionally, cyclo-polycondensation is a simple and direct route for obtaining cation-functioned polymer membranes (Figure 3c). The obtained polymeric aromatic ionenes avoid the heteroatom linkages or electron-withdrawing groups in the backbone. A representative example is the spiro-ionene reported by Jannasch et al., which has no degradation detected after 1800 h alkali aging in 1 M KOD/D_2_O at 80 °C.^45^ Due to its water-soluble characteristics (caused by high IEC), future practical application needs further effort. Comprehensively considering the membrane preparation method and membrane performance, the PBI has more application potential. However, the application of cation-functionalized PBI in alkaline media needs to be improved. Fortunately, due to the amphoteric nature of imidazole, the acid- or alkali-doped PBI membranes have attracted increasing research interest in high-temperature fuel cells, flow batteries, and water electrolyzers.

### 2.4 Polyolefin-Based Alkaline Membranes

Polyolefin is a potential candidate for scalable membrane production. The past decade has seen the progress of multiple backbones, mainly including polystyrene (PS), poly(styrene-ethylene-*co*-butylene-styrene) (SEBS), polynorbornene (PNB), and polyethylene (PE). Generally speaking, their polymer backbones are linear and flexible, thus allowing the membrane to exhibit greater water uptake.

**Figure 4:**
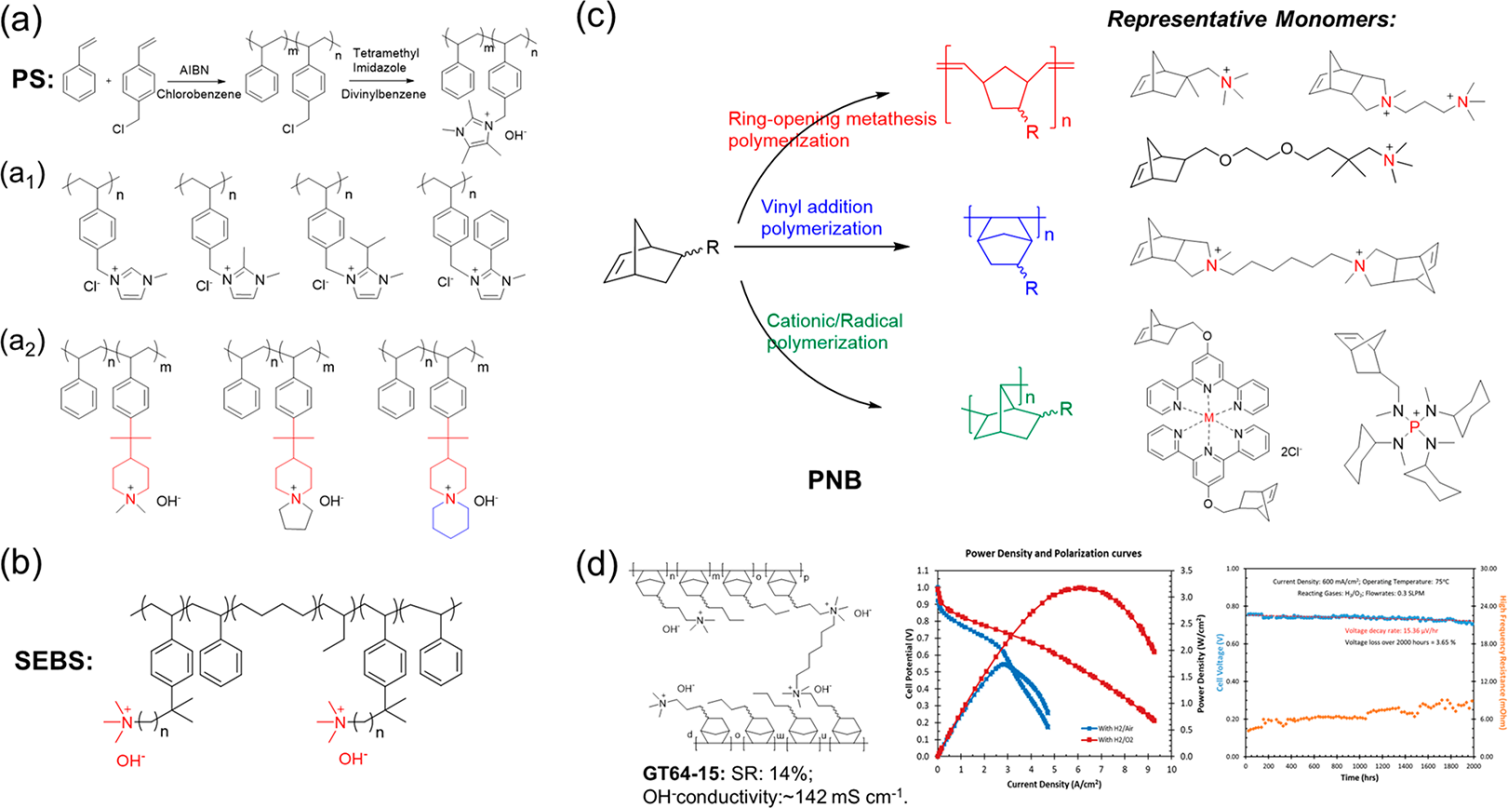
(a) Free radical addition polymerization to prepare Sustanion and representative chemical structure of PS-based alkaline membranes.^46,48,50^ (b) Representative SEBS-based alkaline membranes.^51^ (c) Polymerization methodologies of representative norbornene monomers to prepare PNB.^52−56^ (d) Chemical structure of PNB-based alkaline membranes and related H_2_/O_2_ fuel cell performance.^57^ Reproduced with permission from ref (57). Copyright 2020 John Wiley and Sons.

The represented commercial product is Sustanion produced by the Dioxide Materials company^46^ (Figure 4a). The simple BTMA membrane reported by Sun et al.^47^ showed inferior properties. The rigidity nature of PS restricted the improvement of mechanical properties. To improve the stability of PS-based membrane, more focus was put on anion groups. As depicted in Figure 4a_1_, Yan et al.^48,49^ systematically studied the stability of different cationic groups based on the PS backbone. The results demonstrated that the imidazolium cations could be stabilized by C_2_- and (or) N_3_-substituents in alkaline solution. Additionally, imidazolium cations with strong electron-donating groups at the N1(3) position are more favorable when compared with those at the C_2_-position. Alkali-stable mono- and spirocyclic piperidine-based cations were introduced to the PS backbone using superacid-mediated Friedel–Crafts alkylation^50^ (Figure 4a_2_). So some strategies, including copolymerization with softer monomers and cross-linking, can be carried out. Typically, the SEBS triblock copolymers in Figure 4b are developed because of their excellent flexibility and film-forming properties.^51^ Generally, the SEBS-based membrane outperformed PS membranes in terms of conductivity owing to the well phase separation. However, the poor solubility in common solvents limits their application.

Research on PNB greatly enriched the types of polyolefin. As presented in Figure 4c, the PNB can be obtained from three different polymerization methodologies including metathesis, addition, and isomerization mechanisms from the Norbornene and its derivatives.^58^ Coates et al.^52^ first used ring-opening metathesis polymerization (ROMP) to fabricate PNB-based alkaline membranes bearing trimethylammonium moieties. The absence of β-hydrogen atoms in membrane structure prevents degradation by Hofmann elimination and improves ammonium group stability. In a follow-up report, a series of PNB-based structures that adopt cross-linking, altering tethered cation groups and other strategies, have been developed to improve membrane properties.^55,56,59^ Hickner et al.^53,54^ reported a series of metal-cation-based AEMs, featuring bis(norbornene) ruthenium cobalt or nickel complexes, which enriched the structural types of AEMs. However, a large number of double bonds in backbones resulted in poorer alkaline stability.^60^ Additionally, the PNB synthesized by ROMP has a low *T*_g_ range (30–70 °C), which may result in poor thermomechanical stability of membranes. Another vinyl addition polymerization method enabled the PNB with an increased thermostability due to the high glass transition temperature (*T*_g_ > 300 °C).^61,62^ The following developed XL5-PNB-X_34_-Y_66_ membrane with 5 mol % cross-linker showed the highest conductivity (198 mS cm^–1^) and excellent alkaline stability for 1000 h (1 M NaOH) at 80 °C.^63,64^ Encouragingly, the PNB-based fuel cell performance (PPD of 3.2 W cm^–2^ and durability of 2000 h) displayed in Figure 4d is the highest recorded.^57^ Still, the PNB-based membrane has inferior mechanical properties without reinforcement. The PNB synthesized by radical or cationic polymerization has the problems of low yield, olefin residue, and inconsistent product structure, so it has not been thoroughly investigated in the alkaline membranes field.^58^

**Figure 5:**
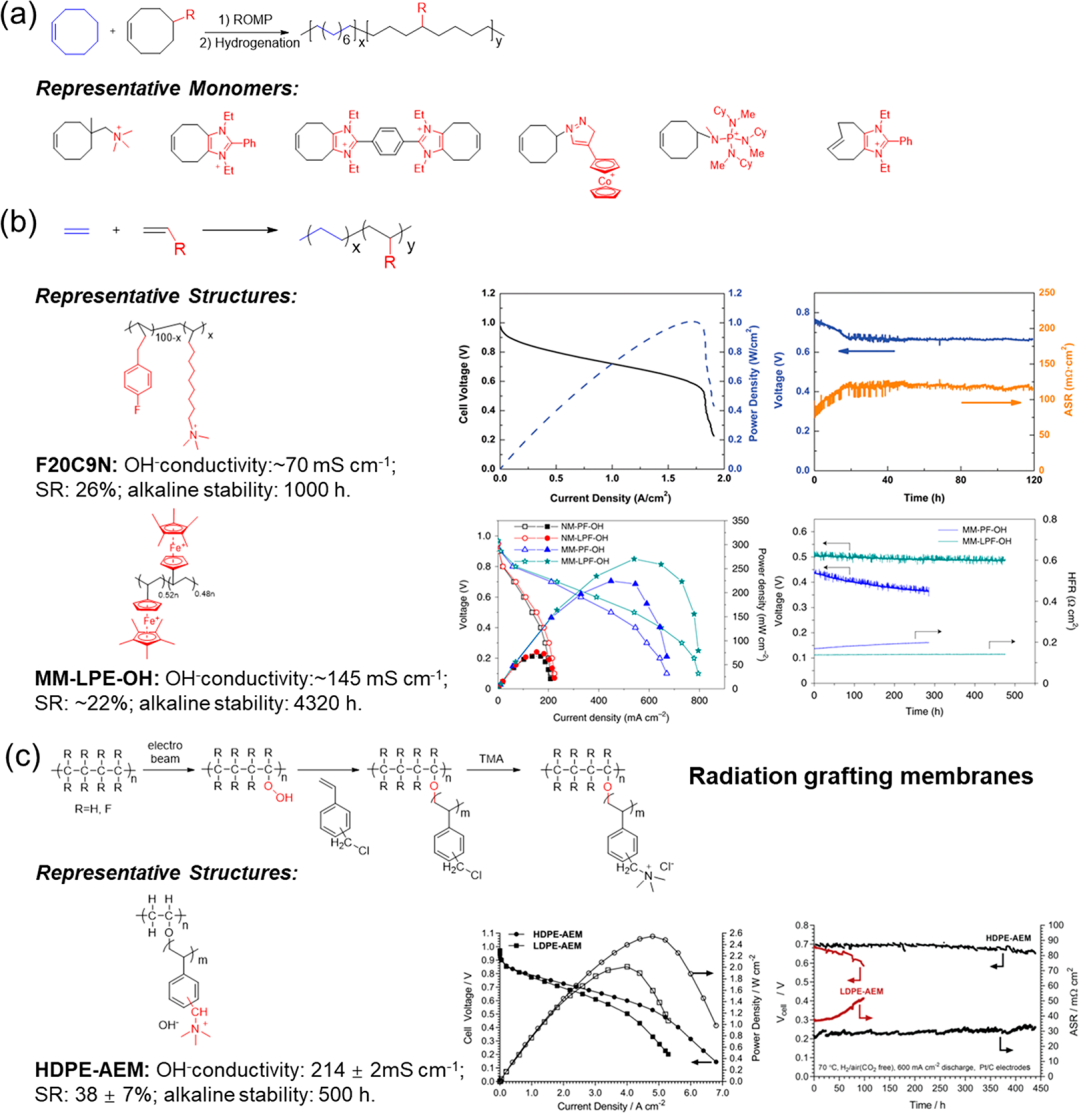
(a) ROMP to prepare PE by representative cyclooctene monomers. (b) General reaction structure formulas for other PE.^60,65,69,70^ The representative F20C9N^67^ and MM-LPH-OH^68^ and related H_2_/O_2_ fuel cell performance. (c) Radiation grafting membranes. The representative HDPE and related H_2_/O_2_ fuel cell performance. Reproduced with permission from refs (67), (68), and (71). Copyright 2019 John Wiley and Sons, Copyright 2022 Springer Nature, and Copyright 2019 Royal Society of Chemistry.

PE is also a common polyolefin backbone used for alkaline membranes. As shown in Figure 7a, the cation-functionalized cyclooctene monomers (tetraalkylammonium, multisubstituted imidazolium, tetrakis(dialkylamino)phosphonium, and cobaltocenium) were adopted to prepare the PE-based alkaline membranes via ROMP.^65,66^ Although promising membrane properties have been achieved, the ROMP synthetic method required the use of noble-metal-catalyzation and hydrogenation reaction, resulting in the complicacy and potential cost of scalable synthesis. On the contrary, the PE-based membrane catalyzed via Ziegler–Natta does not involve precious metal catalyst and has the prospect of industrial synthesis (Figure 5b). The prepared membrane of F20C9N demonstrated excellent OH^–^ conductivity of 91 mS cm^–1^ (80 °C, 100% RH) and alkaline stability (17% conductivity loss after 1000 h in 1 M NaOH, 80 °C). The F20C9N was able to achieve a high-performing and stable operation in fuel cells (Figure 5b).^67^ Recently, the MM-LPH-OH membrane synthesized through free radical polymerization demonstrated the ultralong-record alkaline stability (no detectable conductivity degradation at 95 °C in alkali after 4320 h) after being oriented under magnetic field. Also, the assembled fuel cells demonstrated improved durability (3.9% voltage decreases within 5000 h) under the harshest condition of 120 °C and 40% RH.^68^

Besides functional monomer copolymerization, radiation grafting is a utility method for obtaining functionalized PE membranes (Figure 5c). Tetrafluoroethylene–hexafluoropropylene copolymer (FEP)^72^ and poly(ethylene-*co*-tetrafluoroethylene) (ETFE)^73^ were previously reported as feasible cases to obtain alkaline membranes. But these fluorinated precursor films generally have weakened mechanical properties due to the damage to backbones during the radiation-treated process. Hence, a long-term fuel cell application is hard to maintain due to mechanical constraints caused by swelling stress and membrane rupture. Varcoe et al.^74^ enhanced the mechanical properties of the membrane (TS = 19 MPa, *E*_b_ = 10%) by reducing the radiation doses of the electron beam (from 70 to 30–40 kGy by replacing isopropyl alcohol with water as the dilution solvent) and optimizing the membrane preparation conditions. Further, they found that the commercial availability of hydrocarbon-based polymer precursors has enhanced mechanical strength (TS = 29 MPa, *E*_b_ = 278%) and conductivity (145 mS cm^–1^ at 80 °C, 95% RH) owing to the absence of C–F and a high grafting rate.^75^ The higher anion conductivity and enhanced in situ water transport enable LDPE-AEM (1.45 W cm^–2^) to outperform the ETFE-AEM (1.21 W cm^–2^) in a fuel cell system. Especially, switching the LDPE to mechanically stronger HDPE significantly extends the durability of fuel cells (100 h vs 440 h).^71^

Although numerous polyolefin alkaline membranes have been developed using various polymerization or modification methods, their excellent performance in fuel cells also proves that polyolefin membranes have great application potential. Nonetheless, the mechanical strength of polyolefins at elevated temperatures and their stability against reactive oxygen species are suspicious. Simultaneously, application in other devices needs to be further verified.

### 2.5 Polyaromatic Hydrocarbon-Based Alkaline Membranes

The poly(aliphatic) backbone is prone to decomposition under strong oxidation conditions because the single C–C bond in the main chain is quite unstable.^76^ To pursue more stable polymer backbones, researchers have found that besides the excellent alkaline resistance, the polyaromatic hydrocarbon backbones also have some unique properties, such as low water absorption, high *T*_g_, and good thermal, chemical, and mechanical stability. This section summarizes several typical structures of polyaromatic hydrocarbon backbones and the corresponding synthesis methods.

**Figure 6:**
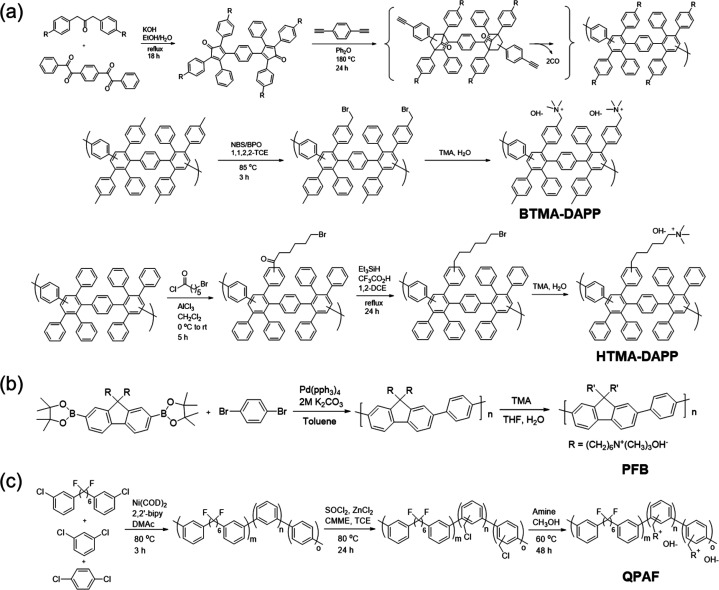
(a) Diels–Alder reaction for preparing HTMA-DAPP membrane.^77,78^ (b) Suzuki coupling reaction^80^ and (c) nickel-catalyzed coupling reaction^82^ for preparing polyaromatic hydrocarbon-based alkaline membranes.

The Diels–Alder reaction is one of the earliest cases of heteroatom-free membranes (Figure 6a) made from all-aromatic polymers.^77,78^ The poly(phenylene) (HTMA-DAPP) membrane reported by Kim et al. exhibited a satisfactory OH^–^ conductivity of ∼120 mS cm^–1^ at 80 °C and alkaline stability (1 M NaOH, 80 °C for 720 h).^78^ However, this kind of membrane has some inherent drawbacks, e.g., time-consuming and complex synthesis, poor solubility, processing difficulty, uncontrollable functionalization location, and greater rigidity. The cross-coupling reaction catalyzed by a transition metal is another critical chemical reaction to build the aromatic C–C linked polymer backbone. Aromatic C–C bonds are usually formed by aromatic halides and corresponding electrophiles with an organometallic catalyst. The Suzuki coupling reaction using aromatic boric acid/borate ester and aromatic bromine compound catalyzed by palladium complex is a common method.^79^ Bae et al.^80^ fabricated the alkaline membrane with the first use of this reaction (Figure 6b). The resultant QA-functionalized polyfluorene membrane showed good conductivity of 124 mS cm^–1^ at 80 °C. The almost identical viscosity measurement before and after the alkali-stable measurement suggested no chain scission, which further verified the excellent chemical stability of synthesized backbone. However, adoption of expensive palladium catalysts economically limits the mass synthesis of materials. By contrast, the nickel-catalyzed coupling reaction is considered a relatively cost-effective and efficient method for polymerization of aryl halides. Miyatake et al.^81−83^ synthesized a membrane composed of perfluoroalkylene and quaternized oligophenylene groups in Figure 6c. However, the need for a large quantity of catalysts and the fact that some monomers cannot be directly commercially available are defects that cannot be ignored in membrane preparation.

**Figure 7:**
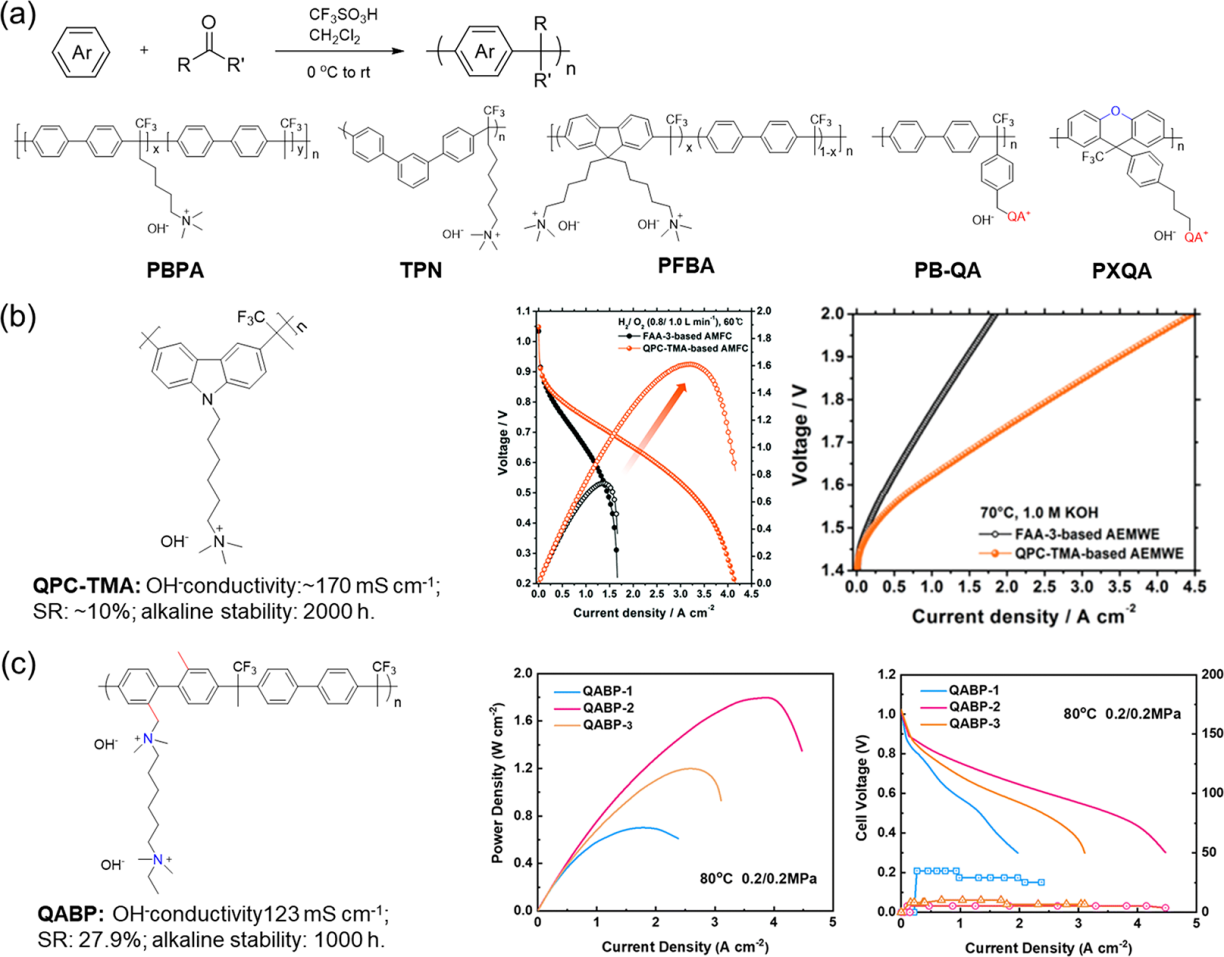
(a) General scheme of acid-catalyzed Friedel–Crafts polycondensations and representative chemical structure.^86−88,91,92^ (b) Chemical structure of poly(carbazole) membrane^89^ and related H_2_/O_2_ fuel cell and water electrolytic performance. (c) Chemical structure of QABP-2^90^ and related fuel cell performance. Reproduced with permission from refs (89 and 90). Copyright 2020 and 2022 Royal Society of Chemistry.

As previously reported, complex synthetic, harsh reaction conditions and high fabrication cost (noble metal catalyst such as Pd was used) were involved in membrane preparation. Hence, progress in developing simple membrane preparation methods and high-performance membrane materials is still urgently required. The Friedel–Crafts polycondensations between super electrophilic ketone groups and electron-rich aromatic compounds (Figure 7a) have overwhelming dominance in chemical reactions, featuring simple steps, flexible reaction conditions, and high efficiency.^84,85^ The resulting aryl carbonyl copolymers are a linear material with high molecular weight, narrow molecular weight distribution, and excellent alkaline stability. The poly(biphenyl alkylene) membrane presented is the first example reported by Bae et al.,^86^ which confirmed the successful migration of this method in the membrane preparation field and opened a new route for membrane preparation. Since 2015, a series of alkaline membranes have been developed, roughly divided into several categories: poly(benzene alkyl), poly(fluorene), poly(carbazole), poly(xanthene)s poly(aryl isatin), and poly(aryl piperidinium). Herein, the membrane (TPN) synthesized through *m*-terphenyl and 7-bromo-1,1,1-trifluoroheptan-2-one and functionalized with quaternary amine has been commercialized and named Orion-TM1.^87^ The membrane displayed promising conductivity of 112 mS cm^–1^ (80 °C) and 5.6% IEC decline after 720 h (1 M NaOH, 95 °C). Other monomers, such as fluorene, carbazole, and 2,2′-dimethylbiphenyl, have also been proven to obtain high-performing alkaline membranes. For instance, the PFBA membrane exhibited a conductivity of 145 mS cm^–1^ (80 °C) and alkaline age of more than 1440 h.^88^ As depicted in Figure 7b, the reported poly(carbazole) membrane with desirable OH^–^ conductivity and alkaline stability has demonstrated excellent performance (1.61 W cm^–2^) in both fuel cells and water electrolyzers (3.5 A cm^–2^ at 1.9 V with 1 M KOH fed).^89^ Attributed to the high molecular weight characteristic of obtained poly(benzene alkyl) membrane, it can be easily prepared into ultrathin membrane materials, thus significantly reducing the mass transfer resistance. Figure 7c shows the performance of fuel cells assembled with ultrathin QABP-2 membrane,^90^ which is up to 1.8 W cm^–2^. Despite great improvements in membrane conductivity and cell performance of poly(benzene alkyl)-based membranes, cell durability is still unsatisfactory. One issue is that most of the poly(benzene alkyl)-based membranes functionalized by trimethylamine face degradation risk at elevated temperatures (≥80 °C).

**Figure 8:**
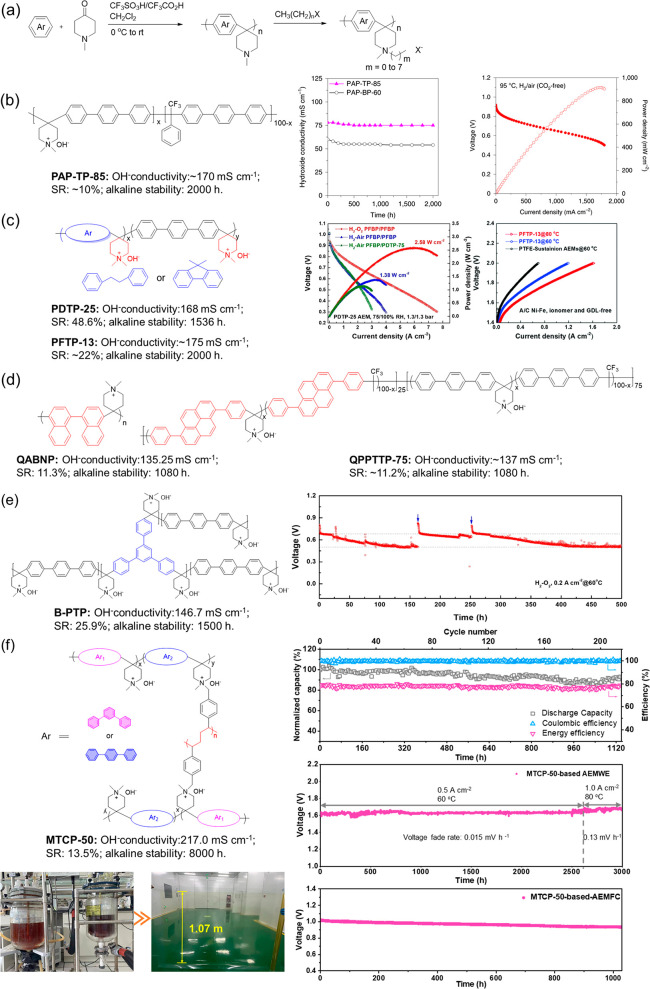
(a) Synthesis route of poly(arylene piperidine). (b) Chemical structure and alkali stability of PAP-TP-85^94^ and related H_2_/Air fuel cell performance. (c) Chemical structure of poly(arylene piperidinium)^95−97^ and related H_2_/O_2_ fuel cell and water electrolytic performance. (d) Representative membrane structure with interactions between molecular chains.^98,99^ (e) Chemical structure of branched poly(terphenyl piperidinium)s and its durability in fuel cells.^100^ (f) Chemical structure of cross-linked MTCP-50 and its durability in neutral aqueous organic redox flow batteries, water electrolyzers, and fuel cells (open-circuit voltage durability).^101^ Reproduced with permission from refs (94), (95), (97), (100), and (101). Copyright 2019 Springer Nature, Copyright 2020 John Wiley and Sons, Copyright 2021 Royal Society of Chemistry, Copyright 2021 John Wiley and Sons, and Copyright 2023 Springer Nature.

The pursuit of more stable cation membranes drove the development of poly(aryl piperidinium) (PAP) (Figure 8a). In 2017, a poly(arylene piperidinium) membrane was first provided by Jannasch et al.^93^ The introduction of this stable cycloamine cationic group enabled membrane stability up to a higher level. The research results showed ring-opening elimination was the main degradation approach of piperidinium groups. The grafted side chains in piperidinium are detrimental to membrane stability and increase with the side chain length. Regrettably, these membranes lack a practical application. Subsequently, the PAP-TP-*x* fabricated by Yan et al.^94^ demonstrated the application potential in fuel cells (Figure 8b). PAP-TP-85 achieved the dramatic conductivity of 78 to 193 mS cm^–1^ in the temperature range from 20 to 95 °C. Additionally, only 3% IEC decreased, demonstrating high alkaline stability. The assembled fuel cell membrane electrode exhibited a high PPD of 0.92 W cm^–2^ and extended durability of 300 h with low Pt electrocatalyst loading and H_2_ and CO_2_-free feed. More surprisingly, the good high-temperature resistance of PAP-TP-85 made the fuel cell’s operating temperature break through 80 °C and reach 95 °C. Different polymerization monomers produced unexpected effects on the membrane properties and cell performance. The PDTP-*x* developed by Lee et al. in Figure 8c displayed preferable phase separation and demonstrated exceptional PPD of 2.58 W cm^–2^ in H_2_/O_2_ fuel cell configuration.^95^ Further, the PFTP-*x* obtained by the use of rigid fluorene showed high water diffusivity on the basis of high conductivity and durability, which contributed to the improved mass transfer efficiency. It enabled the PFTP-*x* membrane to demonstrate excellent performance not only in fuel cells^96^ (PPD of 2.34 W cm^–2^, durability of 200 h) but also in the water electrolyzers^97^ (current density of 7.68 A cm^–2^ at 2.0 V with 1 M KOH fed, durability more than 1000 h). Additionally, some novel monomers such as 1,1′-binaphthalene and pyrene were exploited to enhance the interaction between molecular chains and improve dimensional stability by regulating the polymer geometry configuration without sacrificing conductivity and alkali stability^98,99^ (Figure 8d). Most notably, the adopted cross-linking strategy enables fabricated alkaline membranes to exhibit extending operating durability. As shown in Figure 8e, the branched poly(terphenyl piperidinium)s-based fuel cells can operate stably for over 500 h without membrane damage.^100^ Besides, our group recently provided a type of cross-linked MTCP-50 membrane, as shown in Figure 8f.^101^ The membrane demonstrated exceptional durability in neutral aqueous organic redox flow batteries (negligible permeation of redox-active molecules over 1100 h), water electrolyzers (durability over 3000 h), and fuel cells (open-circuit voltage durability test over 1000 h). This outstanding durability is inseparable from the membrane ex situ stability, which shows 94.3% conductivity retention even after 8000 h alkali aging. The molecular design is ingenious, in which ether-free backbones and piperidinium cation were coupled, and the monomer design adopts the flexible and twisted M-terphenyl to mitigate the conformational distortion of the ring. Then, the cross-linking further strengthens the membrane stability. Therefore, considering the influence of each part comprehensively and improving it is beneficial to improving the membrane stability. What is more exciting is that this membrane can be scaled up in production, which will promote the development of relevant energy technologies.

**Figure 9:**
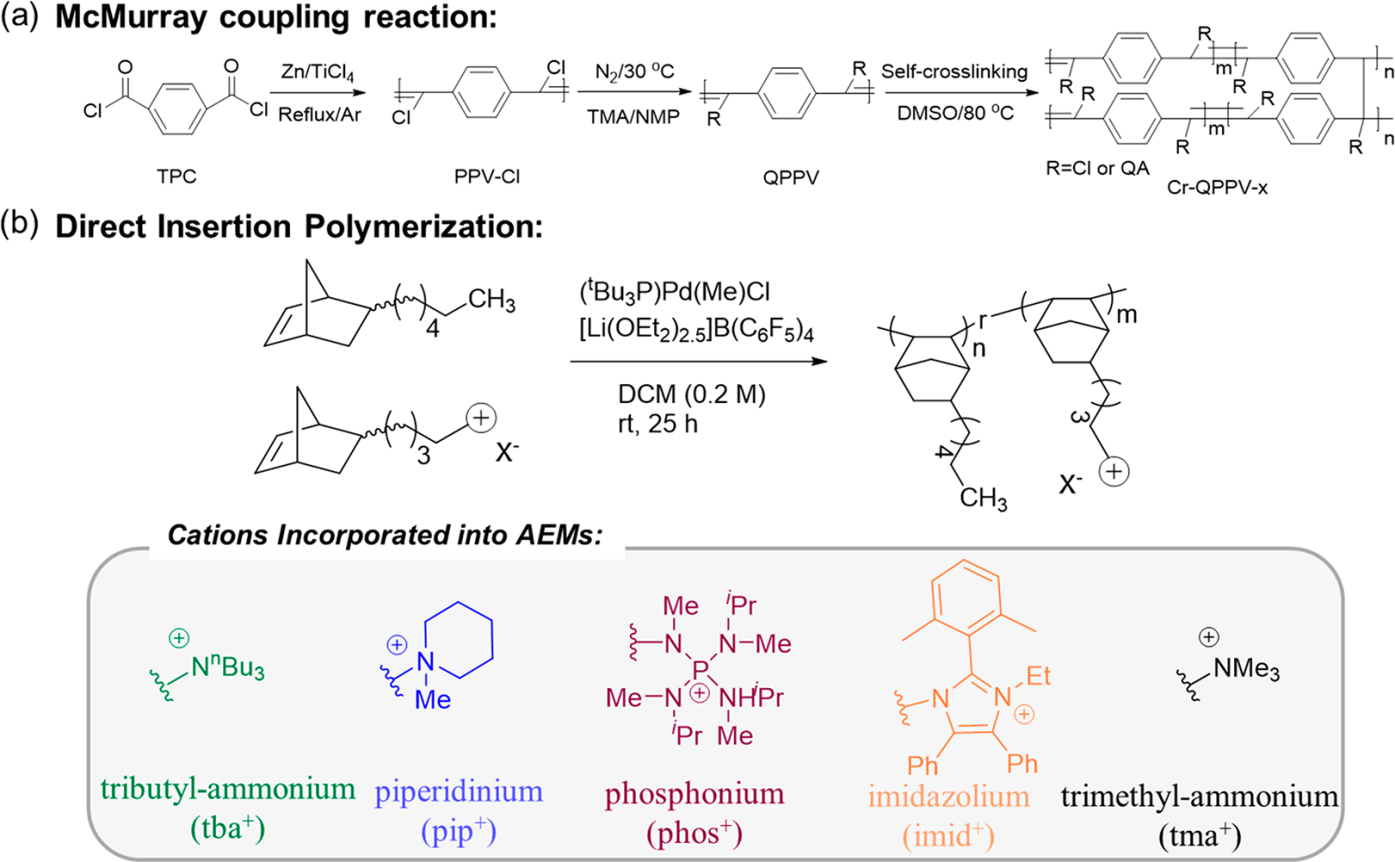
(a) McMurray coupling reaction for preparing alkaline membranes.^102^ (b) Direct insertion polymerization method for preparing alkaline membranes.^103^

Most currently developed high-performance alkaline membranes are based on the superacid-catalyzed Friedel–Crafts reaction. But it has to be pointed out that the production of a large amount of waste acid during membrane synthesis will form a restrictive relationship between polluting the environment and increasing the membrane manufacturing cost. Hence, exploring simpler, more efficient, and economical membrane synthesis methods requires much attention in the membrane field. Our group recently reported a facile, gentle, and low-cost way, called the McMurray coupling reaction (Figure 9a), for the preparation of high-performance alkaline membranes.^102^ Furthermore, Fors et al.^103^ reported a direct coordination–insertion polymerization method of ionic monomers, with the benefits of efficiency and general applicability (Figure 9b). This direct insertion polymerization method avoids the postpolymerization modification process in the traditional synthesis method and allows facile access to a broad range of materials. These successful attempts might provide ideas for membrane production and trigger researchers to explore more economical and simpler synthetic routes. For instance, the Scholl reaction is the coupling reaction between aromatic rings; with the help of Lewis acid (AlCl_3_ and FeCl_3_) instead of noble metal catalyst it can effectively eliminate an H atom on the benzene ring and produce a new C–C bond.^104−106^ This reaction has shown encouraging advantages in the preparation of microporous polymers, so it might have great potential in membrane synthesis.

Overall, polyaromatic hydrocarbon-based alkaline membranes are the most popular category, especially those synthesized via Friedel–Crafts polycondensation. The comprehensive performance improvement in conductivity, stability, and mechanical properties brings great hope to the the terawatt-scale deployment of electrochemical energy technologies. In the future, we should pursue the green economic membrane preparation route on the basis of guaranteed performance.

### 2.6 Relationship between the Polymer Backbone and Membrane Properties

Abundant research efforts have been dedicated to the design and synthesis of alkaline membranes. The alkaline membrane fabrication was shifted from early modification of traditional engineering plastics to molecular structure design starting from monomers currently. Hence, multiple polymer backbones and polymerization methods have been exploited accordingly. Table 1 gives a brief summary in terms of polymerization strategies. We further summarize the relationship between polymer backbone and membrane properties, including conductivity, alkaline stability, and mechanical properties, which have important implications for the future design of polymers.

**Table 1:** Summary of Different Polymerization Strategies for Backbones

		Summarize	
Synthetic method	Examples	Advantages	Disadvantages
Nucleophilic substitution reaction	Partially fluorinated poly(arylene ether)	*Mild reaction condition	*Contains aryl ether groups
Dehydration cyclization polycondensation	Poly(arylene imidazolium)	*High strength, high-temperature resistance	*Stability of the imidazole ring is inferior
Cyclo-polycondensation	Spiro-ionene	*Mild reaction condition	*High IEC leads to solubility in water
Free radical addition polymerization	Polyolefin	*Low cost	*Difficulty controlling structure of the product
		*Reaction maturation	
Ring-opening metathesis polymerization	Poly(norbornene)	*Mild reaction condition	*Low *T*_g_ range
Vinyl addition polymerization		*Good chemical and stereoselectivity	*Requires expensive catalysts
		*Mild reaction condition	
Cationic polymerization		*Fast polymerization rate	*Low yield
			*Olefin residue and inconsistent product structure
Diels–Alder polymerization	Poly(phenylene)	*Metal-free polymerization	*Complex reaction steps
		*Aryl ether-free backbone	*QA precursor incorporation is limited
			*Poor solubility and processability
Pd-catalyzed coupling reaction	Poly(fluorene)	*Mild reaction condition	*Requires expensive Pd catalysts
Ni-catalyzed coupling reaction	Poly(perfluoro alkylene phenylene)	*Less expensive nickel catalysts	*Requires a large quantity of catalysts
	Poly(fluorene phenylene)		*Some monomers cannot be directly commercially available
Acid-catalyzed Friedel–Crafts polyhydroxyalkylation	Poly(benzene alkyl)	*Simple and efficient reaction	*Requires dangerous superacid
	Poly(arylene piperidinium)	*Excellent solubility in polar aprotic solvents	
McMurray coupling reaction	π-Conjugated Polybenzene	*Low cost	*Poor solubility after membrane formation
Direct coordination–insertion polymerization	Poly(norbornene)	*Efficient and generally applicable	*Requires expensive Pd catalysts
		*Avoids postfunctionalization	

Membrane conductivity is highly related to its structure. Most of the previous research work focuses on the modification of side-chain engineering. That is, increasing the number of cation groups or altering the flexibility of the side chains to increase the polarity difference between the polymer backbones and the side chains and to promote the movement of the side chains, so the ion transport channel can be built and thus the conductivity can be improved. Some recent reports have shown that in addition to the hydrophilic side chain, the hydrophobic polymeric backbones also significantly affect the membrane properties, especially the emerging PAP backbone composed of completely hydrophobic groups, showing high conductivity, alkaline stability, and mechanical properties. Xu et al.^107^ used molecular dynamics simulations to provide a mechanism for the influence of backbone hydrophobicity on membrane transport properties at the molecular level. Three representative polymer backbones (PAES, PPO, and PAP) with different hydrophobicity were selected and compared. The theoretical simulation results demonstrated that the hydrophobic backbone is conducive to forming more considerable and connected water phases. Meanwhile, the hydrophobic backbone has a weak interaction with water molecules, which makes the water and hydroxides no longer bound by the solvation shell of the backbone segments. The hydrophobic backbone repels the water and hydroxides and enters the hydrophilic phase to form connected ion channels. Therefore, the hydrophobic backbone is favorable for anion conductivity. This conclusion is also supported by relevant experimental evidence. The conductivity of QABP-2 membrane (hydrophobicity ether-free backbone) displayed ∼1.24 times higher than that of BQAPPO (less hydrophobicity ether backbone) under the same IEC value and side chains.^90^ These results further explain why the conductivity of aryl-ether-free alkaline membranes is generally higher than aryl-ether-containing alkaline membranes.

Membrane stability is jointly affected by the skeleton, anion groups, and linkers. In terms of the influence of the backbone on membrane stability, most of the poly(arylene ether)-based alkaline membranes exhibited a great decrease in conductivity within 1000 h, even at lower temperatures (60 °C). Certainly, the membrane stability is greatly improved after removing polar electron-withdrawing groups and unstable linked bonds. Polyolefin membranes also showed some noticeable performance degradation under high temperatures and alkaline conditions. In particular, the oxidation stability is doubtful. The overall stability of aryl ether-free polyaromatic membranes is reported to outperform polyolefin membranes. However, it is worth noting that the membrane stability is different even in the same class of alkaline membranes. Take PAP as an example, the stiff backbone may increase the conformational distortion of the rings and lead to reduced alkaline stability.^93^ A report by Bae et al.^87^ demonstrated that the IEC loss of m-TPN1 (∼0.9%) with flexible *m*-triphenyl is lower than p-TPN1(∼1.8%) with rigid *p*-triphenyl after the same alkali aging treatment. Careful structural design is therefore required to realize the desired alkaline stability. Based on the existing stable structure, further investigation into the relationship between polymer conformation and alkaline stability may provide a deeper understanding of the development of alkaline membranes.

Mechanical integrity is one of the most critical prerequisites for alkaline membranes regarding membrane electrode assembly fabrication, handling, assembling, and operating. Robust and tough alkaline membranes are required due to mechanical and swelling stress. Moreover, the membranes must have a certain elasticity (elongation) to prevent crack formation. The molecular weight of the polymer chains mainly determines the membrane’s mechanical properties. And it is also affected by the water content. Generally, the rigidity and flexibility of the polymer backbone positively affect tensile strength (TS) and elongation at break (*E*_b_), respectively. For instance, the rigid nature of polyaromatic hydrocarbon-based alkaline membranes enables it to have higher TS (generally >30 MPa).^94,96,101^ The typical polyolefin-based alkaline membranes usually display extraordinary *E*_b_ (generally >100%).^108,75^ For poly(arylene ether)-based alkaline membranes, the TS is generally <30 MPa while *E*_b_ < 20%.^109,110^ A good balance between TS and *E*_b_ should be considered in molecular design to obtain the optimum mechanical properties of the material. Another thing that needs to be accounted for in addition to tensile testing to evaluate the membrane mechanical properties is compressive stress, which is the main force the membrane is subjected to in the device. Hence, future attention should be focused on assessing how the membrane deforms during extrusion, such as using nanoindentation measurements to evaluate the hardness of the membrane.

In summary, precisely manipulating molecular topology enables the hydroxide conductivity to exceed 140 mS cm^–1^ and even up to 200 mS cm^–1^. Furthermore, with the profound dissection of the membrane degradation path in the alkaline environment, years of research have provided various strategies, such as alkyl spacer introduction, electron-withdrawing and benzylic position elimination, block structure and cross-linking design, microphase separation construction, ether bond avoidance, and alkali stable cation execution. In particular, the newly developed ether-bond-free aryl backbone coupled with stable N-heterocyclic ammonium groups makes high conductivity and alkali resistance stability possible. The great efforts of researchers in the membrane community gave birth to several commercial membrane products and promoted the rapid development of membrane technology. Table 2 summarizes the membrane brand name, manufacturing location, synthesis methodologies, and related physicochemical properties. It can be seen that low-cost, scalable production methods for achieving high-performing membranes remain scarce.

**Table 2:**
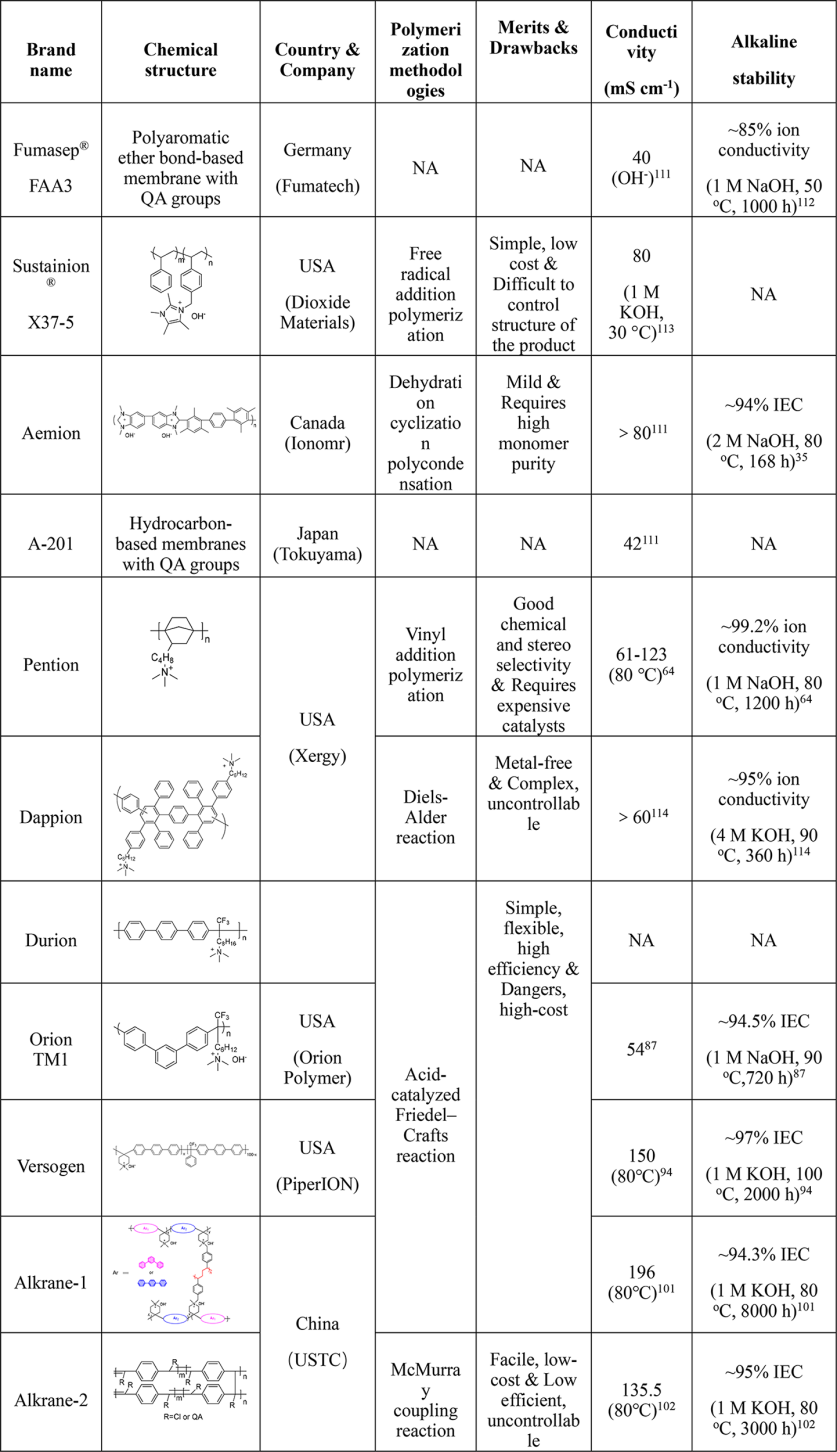
Commercial Alkaline Membranes and Their Reported Properties^a^

a Refs (111−114).

Up to now, the fuel cell performance can easily exceed 1 W cm^–2^ attributed to the fast development of high-performing alkaline membranes, which has been equal to or exceeded PEM fuel cells. Nonetheless, the fuel cell durability is generally lower than 500 h. It is far from the U.S. DOE goal (durability exceeds 1000 h at 0.6 A cm^–2^). The durability of reported electrolytic cells is also generally within 1000 h with the current density below 1 A cm^–2^. Overcoming this challenge requires an in-depth comprehension of the association between membrane stability and cell durability. Hence, in the following section, we analyze several aspects that are most likely to restrict membrane stability and cell durability.

## 3 Membrane Stability Investigation

### 3.1 Alkaline Stability

Encouragingly, great achievement has been made in the development of alkali-stable membrane materials over the past few years. The protocols and methods for evaluating membrane stability are critical. Currently, there is still no uniform standard and protocol. Testing conditions of alkaline stability are subjective and vary from one study to another (concentration: 1–10 M aqueous NaOH or KOH solution; temperature: rt–100 °C), making direct comparisons between different membranes difficult. The common approach to evaluate the ex situ membrane stability is monitoring IEC or conductivity change over immersion time in aqueous alkali. However, assessing alkaline stability solely from changes in conductivity does not seem to correspond accurately to the chemical degradation of tethered cation groups. Because the change in water uptake or morphology experienced during the alkali aging of the membrane can also alter the conductivity.^115^ Therefore, the most prudent approach is not to rely on one method. A comprehensive analysis of IEC, conductivity, mechanical properties, and water absorption behavior of alkali-aged membranes can further enhance the understanding of limiting factors on stability. Besides, with the development of more alkali-stable membranes and considering closer to the actual operating environment, new ex situ alkaline stability test protocols should be developed.

### 3.2 Oxidation Stability

Notably, in addition to alkali stability, attention should be paid on the oxidation stability. Several literature reports have revealed that membrane degradation occurs under the attack of free radicals (mainly superoxide and hydroxyl radicals).^116−118^ Ramani et al.^117^ used in situ fluorescence techniques to detect the presence of superoxide radicals during anion-exchange membrane fuel cell (AEMFC) operation. Kruczała et al.^116^ clearly displayed the formation and existence of radicals in alkaline membranes during AEMFC operation using an electron paramagnetic resonance (EPR) spectrometer. Ramani et al.^119^ found that membrane degradation preferentially occurred near the oxygen evolution electrode during anion-exchange membrane water electrolyzer operation. All these research results demonstrated that in the existence of oxygen and OH^–^ ions, superoxide and hydroxyl radicals could both be spontaneously generated within the AEMs. The radicals caused membrane backbone degradation and made the membrane thinner. Hence, the oxidation degradation is an emerging concern. At present, Fenton’s reagent is a widely adopted tool for oxidation stability evaluation.^120,121^ But it should be pointed out that this tool is not very suitable for assessing the oxidation stability of alkaline membranes used in an alkaline environment. Because it only supports hydroxyl and hydroperoxyl radicals under acidic conditions, while in alkaline environment, more hydroxyl and superoxide radicals are generated. Perhaps more mature testing protocols should be developed in the future to demonstrate the chemical stability of AEMs.

### 3.3 Mechanical and Dimensional Stability

Apart from chemical degradation, physical degradation also cannot be ignored in actual applications. Mechanical integrity is the most important prerequisite for polymerelectrolyte membranes regarding membrane electrode fabrication, handling, assembling, and durability. Generally, membranes face the physical degradation risk. Membrane creep and microcrack fracture are two common problems.^76^ The assembled membrane in the device undergoes time-dependent deformation under the action of heat and stress (compressive force). This deformation will cause the membrane to become thinner, and after long-term operation, the membrane is more prone to permanent deformation, eventually leading to cell failure. The microcrack failure through the dimensional change caused by humidity and temperature change is also the predominant failure mechanism of membrane materials. Therefore, robust membranes are required to bear mechanical and swelling stress. Moreover, the membrane must have a certain elasticity (elongation) to avoid the formation of cracks.

This section briefly discusses the possible degradation risks of membranes during operation in devices. The failure mechanism of the membrane varies with the electrochemical equipment and operating conditions (temperature, humidity, current density, etc.). Moreover, the membrane failure is not a single factor. At present, most researchers use NMR, IR, and other methods to confirm the membrane structural integrity by dissecting membrane electrode assembly. The in situ detection technology has not been widely used to observe the real-time membrane failure process in service.

## 4 Membrane Application Extension

**Figure 10:**
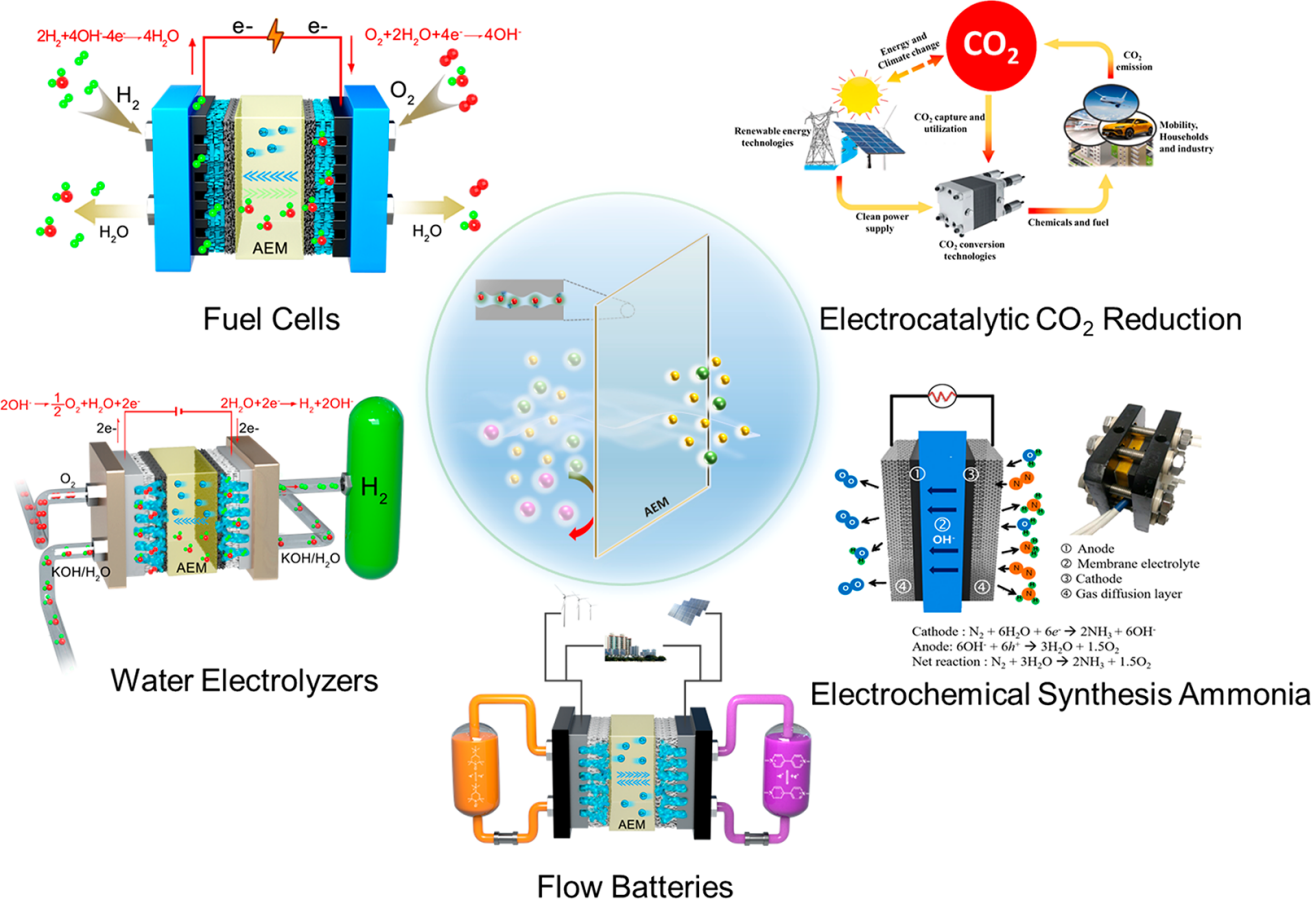
Schematic diagram of the alkaline membranes applied in electrochemical energy devices. Reproduced with permission from refs (101), (122), and (123). Copyright 2023 Springer Nature, Copyright 2020 Elsevier, and Copyright 2017 American Chemical Society.

Recent concerns about sustainability and alternative energy have driven an increasing trend in the research of new energy equipment. Alkaline membrane-based energy technologies offer us a low-cost way of sustainable energy storage and conversion. We herein briefly present alkaline membranes’ potential and promising application in current and future energy technologies (Figure 10).

Currently, fuel cells and water electrolyzers are two mainstream application technologies of alkaline membranes. Therefore, the membrane structure evolution is mostly carried out around these two technologies. In these two processes, the membrane acts as a barrier between cathode and anode and conducts OH^–^ ions. The membrane conductivity is highly correlated with the energy output efficiency of the fuel cells and the electrolytic efficiency of the electrolyzer cells. Meanwhile, the membrane stability determines the service life of the device. Thus, membranes with high OH^–^ conductivity, excellent chemical and mechanical strength, and lower gas permeability are ideal materials. Benefiting from the development of high-performance alkaline membranes, the PPD of H_2_/O_2_ fuel cells has increased from the original 0.055 W cm^–2^ to the current peak of 3.4 W cm^–2^. And the longest durability record is about 2000 h. Moreover, the reported high current density and longest durability (12 000 h) of alkaline membrane-based water electrolyzers as well as the commercialization of Enapter give us increasing confidence to build a more sustainable society in using hydrogen technology.

In addition to these two technologies, some emerging technologies also show a huge demand for alkaline membranes. For instance, flow batteries are regarded as a hopeful large-scale energy storage technology to accommodate the fluctuating and intermittent nature of renewable energy. Herein, the membrane serves to transport anions and acts as an electrolyte separator to prevent electrolyte molecules from crossing over. Therefore, the highly selective anion transport and electrolyte solvent resistant membranes are two keys to ensure high efficiency and stable operation in flow batteries. Recently, the hot research turned to electrocatalytic CO_2_ reduction (CO_2_RR). This technique converts the waste CO_2_ to valuable chemicals and fuels, which is an attractive approach to address carbon recycling and simultaneously realize renewable energy storage. The alkaline membranes act as charge carrier conductors and separators. The poor CO_2_ reduction result (hydrogen evolution reaction is preferred over the CO_2_RR in acidic media) in PEM-based CO_2_RR compelled the exploration of alkaline membrane-based CO_2_RR. Currently, despite properties of available alkaline membranes that have been reported in the context of fuel cells and water electrolyzers, no protocols or metrics have been established for alkaline membrane CO_2_RR. Generally, high anion conductivity, chemical stability, good solvent resistance (especially alcohol solvents), and selectivity are the crucial parameters for optimal membrane performance.^122^ Moreover, as a low-temperature electrochemical synthesis of NH_3_, the electrochemical synthesis of ammonia driven by sustainable energy is a hopeful alternative to Haber-Bosch as a green way. In the PEM-based ammonia generation device, ammonia will react with PEM owing to its weak base nature, affecting its working life. In contrast, an alkaline system reduces the reactivity of the membrane with ammonia, enabling low-cost construction materials and allowing the use of a wider range of low-cost and active catalysts. For these reasons, alkaline membranes are considered an attractive alternative to PEMs. Although there have been few research results of alkaline membranes-based electrochemical ammonia synthesis so far, membranes with optimized OH^–^ transport and durability are required to achieve higher efficiency. This new technology opens a new application route for alkaline membranes, although there have been few research results so far.

One thing worth noting is that despite properties of available alkaline membranes having been reported in the context of fuel cells and water electrolyzers, no protocols or metrics are established for other alkaline membrane-based technologies. Since different technologies have specific property requirements for membranes, currently available membranes may not fully meet the requirements of the electrochemical device. Thus, designing and reoptimizing membrane structures timely according to performance and feedback is necessary to promote the advance of electrochemical energy technology. Furthermore, strengthening the joint use of multiple technologies will promote the sustainable development of renewable energy, such as the combination of flow batteries–water electrolyzer–fuel cells to achieve the cycle of renewable energy power generation, water electrolyzer to produce hydrogen, and hydrogen to generate electricity. Altogether, driving the development of alkaline membranes-based energy technology, especially the membrane advanced, is urgently desired to satisfy human life and production.

## 5 Conclusion and Outlook

Benefiting from molecular engineering, topological regulation, and new synthesis methods, noteworthy advances have been made at multidimensions of alkaline membranes. The accomplished high anion conductivity (>140 mS cm^–1^), alkaline stability (exceeding 2000 h), and mechanical properties (tensile stress >40 MPa and elongation at break >20%) paint a promising picture for alkaline membrane-related technologies. Based on previous efforts, future development trends of alkaline membranes are discussed as follows.

(1) Regarding chemical stability, the heteroatom linkage-free backbone remains reliable. Stable cationic groups still have room for exploration and progress. Precise design of omnidirectional blocking of ionic group degradation paths will greatly improve membrane durability. A special note is that the membranes with the combination of alkali-stable backbones and cations are not necessarily alkaline-stable unless the polymer structure and linkers are also carefully designed.
(2) For polymerization route, although the acid-catalyzed Friedel–Crafts reaction is an efficient and simple method for giving high-quality polymer, it is detrimental to the environment effectiveness and cost competitiveness due to the vast production of waste acid. Hence, developing new polymerization methods with synthetic feasibility and architecture tunability is still urgently needed for large-scale synthesis.
(3) For application, due to the concerns of membrane oxidation degradation caused during operation, focus on designing materials that show both alkaline stability and oxidation stability is required in future studies. Additionally, reinforced alkaline membranes may be a promising direction for optimizing mechanical properties and durability.
(4) Due to the specific requirements of different electrochemical devices on membrane properties, reoptimizing the structure according to performance feedback is needed.

High-performance alkaline membranes are the pursuit goal in the early stage, research efforts should be aimed at achieving large-scale manufacture with cost competitiveness of advanced membrane materials to meet the the terawatt-scale deployment of electrochemical energy technologies.

## Nomenclature

AEMs: anion exchange membranes
PEMs: proton exchange membranes
PAES: poly(arylene ether sulfone)
PAEK: poly(arylene ether ketone
FPAE: partially fluorinated poly(arylene ether)
PPO: poly(2,6-dimethyl-1,4-phenylene oxide)
NMR: nuclear magnetic resonance
QA: quaternary amine
IEC: ion exchange capacity
RH: relative humidity
PBI: poly(benzimidazolium)
PS: polystyrene
SEBS: poly(styrene-ethylene-*co*-butylene-styrene)
PNB: polynorbornene
PE: polyethylene
ROMP: ring-opening metathesis polymerization
PPD: peak power density
EPR: electron paramagnetic resonance
AEMFC: anion-exchange membrane fuel cell
CO_2_RR: electrocatalytic CO_2_ reduction
SR: swelling ratio (%)
